# The glycosylation sites in RBD of spike protein attenuate the immunogenicity of PEDV AH2012/12

**DOI:** 10.1016/j.virusres.2024.199381

**Published:** 2024-05-02

**Authors:** Gege Zhang, Qi Peng, Shiyu Liu, Baochao Fan, Chuanhong Wang, Xu Song, Qiuxia Cao, Chengcheng Li, Hong Xu, Hongting Lu, Meiying Bao, Shanshan Yang, Yunchuan Li, Jiaxiang Wang, Bin Li

**Affiliations:** a College of Animal Science, Yangtze University, Jingzhou 434025, China; b Institute of Veterinary Medicine, Jiangsu Academy of Agricultural Sciences, Key Laboratory of Veterinary Biological Engineering and Technology, Ministry of Agriculture, Jiangsu Key Laboratory for Food Quality and Safety-State Key Laboratory Cultivation Base of Ministry of Science and Technology, Nanjing, Jiangsu 210014, China; c College of Veterinary Medicine, Nanjing Agricultural University, Nanjing, Jiangsu 210095, China; d Jiangsu Co-Innovation Center for the Prevention and Control of Important Animal Infectious Diseases and Zoonoses, Jiangsu Key Laboratory of Zoonoses, Yangzhou University, Yangzhou, Jiangsu 225009, China; e GuoTai (Taizhou) Center of Technology Innovation for Veterinary Biologicals, Taizhou, Jiangsu 225300, China

**Keywords:** PEDV, RBD, Glycosylation, Reverse genetics, Pathogenicity, Immunogenicity

## Abstract

Porcine epidemic diarrhea (PED) is a highly contagious swine intestinal disease caused by PED virus (PEDV). Vaccination is a promising strategy to prevent and control PED. Previous studies have confirmed that glycosylation could regulate the immunogenicity of viral antigens. In this study, we constructed three recombinant PEDVs which removed the glycosylation sites in RBD. Viral infection assays revealed that similar replication characteristics between the recombinant viruses and parental PEDV. Although animal challenging study demonstrated that the glycosylation sites in RBD do not affect the pathogenicity of PEDV, we found that removing the glycosylation sites on the RBD regions could promote the IgG and neutralization titer *in vivo*, suggesting deglycosylation in RBD could enhance the immunogenicity of PEDV. These findings demonstrated that removal of the glycosylation sites in RBD is a promising method to develop PEDV vaccines.

## Highlights

• Three recombinant PEDVs with abolished glycosylation sites in RBD of spike protein were successfully constructed.
• Removal of the glycosylation sites in RBD of spike protein does not influence the pathogenicity of PEDV AH2012/12.
• RBD glycosylation in spike protein could attenuate the immunogenicity of PEDV AH2012/12.

## 1 Introduction

The family Coronaviridae contains four genera, including alphacoronavirus, betacoronavirus, gammacoroanvirus, and deltacoronavirus (Cui et al., 2019). In 1970s, PED was identified sporadically in various regions of Europe and Asia (Pensaert and de Bouck, 1978). In the late of 2010, a considerable number of PED outbreaks caused by variant PEDV were reported in China, resulting in significant economic losses for the pig industry (Li et al., 2012; Sun et al., 2012; Wang et al., 2013). The clinical manifestations of PED mainly include acute diarrhea, vomiting, dehydration, and high mortality in neonatal pigs (Jung and Saif, 2015).

The genome of PEDV is a single-stranded, positive-sense RNA of approximately 28 kb. It contains at least seven open reading frames (ORFs) in the following order of ORF1a, ORF1b, spike (S), ORF3, envelope (E), membrane (M), and nucleocapsid (N) (Chen et al., 2020; Song et al., 2015). The S protein of PEDV can be further categorized into two subunits, the S1 subunit (1–789 aa) and S2 subunit (790–1383 aa) (Su et al., 2016). The receptor binding domain in the S1 subunit is responsible for interacting with specific cellular receptors for viral attachment (Ji et al., 2022; Reguera et al., 2012). Numerous studies indicated that the PEDV S protein is considered to be a highly glycosylated protein and plays a crucial role in immunogenicity and pathogenicity of PEDV (Qian et al., 2019; Watanabe et al., 2020; Yu et al., 2022). The analysis of N-glycosylation sites predicts that the S protein possess 27 potential N-linked glycosylation sites, two of which are located in the RBD of the S protein. In this study, we generated three recombinant PEDVs, named r12-N514G, r12-N556G, r12-N514+556 G, through substituting serine with asparagine amino acids at aa 514 and 556. The purpose of this study was to investigate the impact of N-glycosylation in the RBD of the S protein on the immunogenicity of PEDV AH2012/12. The biological characteristics of these recombinant viruses in cells and pathogenic to piglets were compared to that of the parental AH2012/12 virus, which provide a reference for the RBD-based PEDV vaccine development strategy.

## 2 Materials and methods

### 2.1 Cell, viruses, and antibody

DMEM (Basal Media, Shanghai, China) supplemented with 10 % fetal bovine serum (FBS) (Tianhang Biotech, Hangzhou, China) was used for culture of Vero cells at 37 °C, CO_2_. PEDV AH2012/12 (GenBank accession number KU646831) was isolated from intestinal samples from piglets suffering severe diarrhea and cultured in DMEM containing 5 μg/ml trypsin (Sigma) and 37.5 μg/ml pancreatin (Sigma) (Fan et al., 2017). PEDV AH2012/12 infectious cDNA clone was constructed and maintained in our laboratory (Peng et al., 2022). PEDV N monoclonal antibody was produced in our laboratory (Fan et al., 2022).

### 2.2 Recombinant BAC plasmid construction

The specific guide RNAs (sgRNAs) were designed to target the RBD region of the PEDV S gene. PCR amplification was performed using sgRNAF1/sgRNAR1 and scaffold oligo as primers, followed by purification of the amplicons using a DNA purification kit (Omega Bio-Tek, Guangzhou, China). The purified amplicons were used as templates for sgRNA transcription by combining them with transcription buffer (NEB, Beijing, China), NTPs (NEB, Beijing, China), RNase inhibitor (TAKARA, Dalian, China), DTT (NEB, Beijing, China), and T7 RNA polymerase (NEB, Beijing, China), and incubating overnight at 37 °C. After the sgRNAs were purified, the recombinant bacterial artificial chromosome (BAC) plasmid harboring the AH2012/12 genome was cut *in vitro* using Cas9 protein (NEB, Beijing, China). After that, the linearized recombinant BAC plasmid was purified, and homologous recombination was performed to clone DNA fragments into the BAC plasmid. Positive clones were screening using colony PCR and sequencing. Primer sequences used in this study are listed in Table 1.

**Table 1:** Primer sequences used in this study.

Primer ID	Sequence (5′−3′)
SgRNAF1	TTCTAATACGACTCACTATAGGCAAGTTCAATTGTAAGCATAGTTTTAGAGCTAGA
SgRNAR1	TTCTAATACGACTCATATAGGCTATGTCCGATCTCAGTCGTTTTAGAGCTAGA
PEDVSN10F	GGTGGTTTCCAACCAACCAT
PEDVSN10R	CAATCTTGACTTGGCCAGAC
PEDVSN10MR	AACAAAAGAATGATCATTAA
PEDVSN10MF	TTAATGATCATTCTTTTGTTCAAATTACTGTCTCTGCTGCTTT
PEDVSN11MR	ATAAAACAGTGAAATGGTAA
PEDVSN11MF	TTACCATTTCACTGTTTTATCAAGTTACAAACAGTTATGGTTA
Scaffold oligo	AAAAGCACCGACTCGGTGCCACTTTTTCAAGTTGATAACGGACTAGCCTTATTTTAACTTGCTATTTCTAGCTCTAAAAC
PEDV-25F	ATCTTCTGGCGTAATTCCACA
PEDV-25R	CACCTTACCATGCACCAAAGT

### 2.3 Generation of the recombinant PEDVs

When Vero cells in 6-well plates reached a confluency of 90 %, 6 μg of BAC recombinant plasmid was transfected into Vero cells using Lipofectamine 3000 (Invitrogen). At 8 hours post transfection (hpt), the cells were washed and replaced with DMEM medium containing 5 μg/ml trypsin (Sigma) and 37.5 μg/ml pancreatin (Sigma), and then cultured in a humid incubator with 5 % CO_2_ at 37 °C. Virus recovery was confirmed by daily observation of cytopathic effect (CPE) appearance under a microscope (Nikon).

### 2.4 Indirect immunofluorescence assay (IFA)

Monolayers of Vero cells in 24-well plates were infected with 0.1 MOI of PEDV. At 24 h post infection, the supernatant was discarded and the cells were fixed using 4 % paraformaldehyde at room temperature for 15 min. Next, the cells were treated with a precooling methanol solution for 10 min. Subsequently, the cells were blocked with 5 % skimmed milk at 37 °C for 1 h. Before adding mouse anti-PEDV N monoclonal antibody (1: 500 dilution), the cells were undergone three rounds of phosphate-buffered saline (PBS) washings. After that, the cells were incubated with goat anti-mouse IgG conjugated with FITC (Beyotime, Shanghai, China) (1: 2000 dilution) for 1 h. Following three washes with PBS, the cells were treated with 0.1 % DAPI (Boster Bio, Wuhan, China) at room temperature for 15 min. Finally, the cells were washed thrice with PBS and the fluorescence distribution was examined under a fluorescence microscope (Nikon).

### 2.5 Viral plaque assay

PEDV strains were serially diluted ten times and then added into Vero cells. The incubation process lasted for 1.5 h at a temperature of 37 °C. Following the infection, we washed the cells twice in DMEM to remove unabsorbed viruses. Subsequently, the cells were covered with 2 ml of and cultured in DMEM containing 5 μg/ml trypsin (Sigma) and 37.5 μg/ml pancreatin (Sigma). The cells were then incubated at 37 °C. After the incubation period, 4 % paraformaldehyde fixative was added, then stained the cells with 0.1 % crystal violet.

### 2.6 Viral growth kinetic analysis

To analyze the growth kinetics of the parental and recombinant viruses, viral stocks were inoculated onto Vero cells in 24-well plates with 0.1 MOI. After incubating at 37 °C for 1.5 h, the supernatant was discarded and washed with DMEM for twice. Each well was added with 500 μl of virus maintenance. Subsequently, the supernatant medium was collected at 12, 24, 36, and 48 h, respectively. Then, the viral titers at each time point were determined using 50 % tissue culture infectious dose (TCID_50_) (Vieyres and Pietschmann, 2013).

### 2.7 Animal experiment

The protocol for all experiments involving animals was granted approval by the Institutional Animal Care and Use Committee of the Jiangsu Academy of Agricultural Sciences (NKYVET 2019–0138). A total of 25 5-day old piglets, which were seronegative for PEDV antibody, were randomly assigned into five groups: rAH2012/12 group, r12-N514G group, r12-N556G group, r12-N514+556 G group, and control group. Each group contains five piglets. The piglets in each group were challenged or mock-challenged with 1 × 10^4.5^ TCID_50_ of indicated virus by oral administration. After challenge, daily clinical observations were conducted and rectal swabs were collected to measure virus shedding using real-time reverse transcriptase quantitative PCR (RT-qPCR) (Zeng et al., 2015). Serum samples were collected for determination of the antibody titer against S PEDV by ELISA and viral neutralizing assay (VN). After euthanasia of the piglets at 21 dpi, intestinal segments were collected and processed for RT-qPCR analysis to determine the viral load in intestines, and were fixed in 10 % neutral-buffered paraformaldehyde for histopathological examinations.

### 2.8 Statistical analysis

The data generated in this study are expressed as mean ± standard deviation (SD) and analyzed with software GraphPad Prism version 7.00. Unpaired Student t tests were used to evaluate the statistical difference between the two groups. Statistical differences between groups were considered as statistically significant when the *P* value was lower than 0.05 (*, *P* < 0.05; **, *P* < 0.01; ***, *P* < 0.001).

## 3 Results

### 3.1 Construction of the recombinant PEDVs

**Fig. 1:**
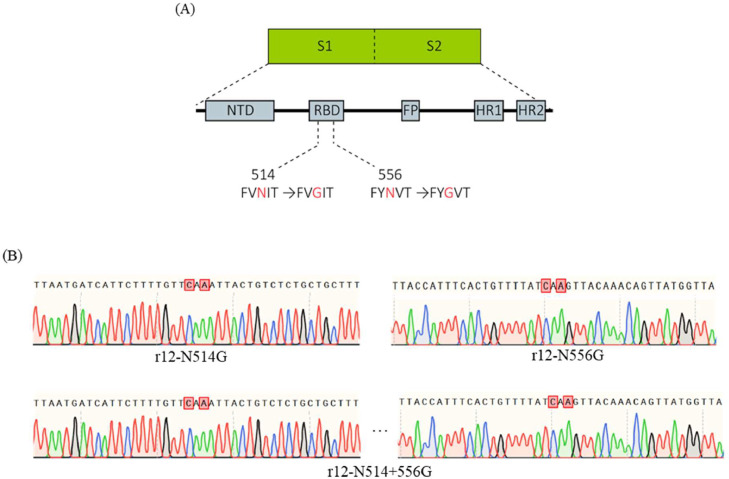
Construction of the recombinant PEDVs. (A). Strategy to construct recombinant PEDVs. BAC-PEDV was cleaved with CRISPR/Cas9 *in vitro*, and then the linearized BAC plasmid was ligated with DNA fragments containing expected mutations. In the amino acid sequence, the mutated amino acids are in highlighted in red. (B). Identification of the recombinant BAC plasmids by sequencing.

**Fig. 2:**
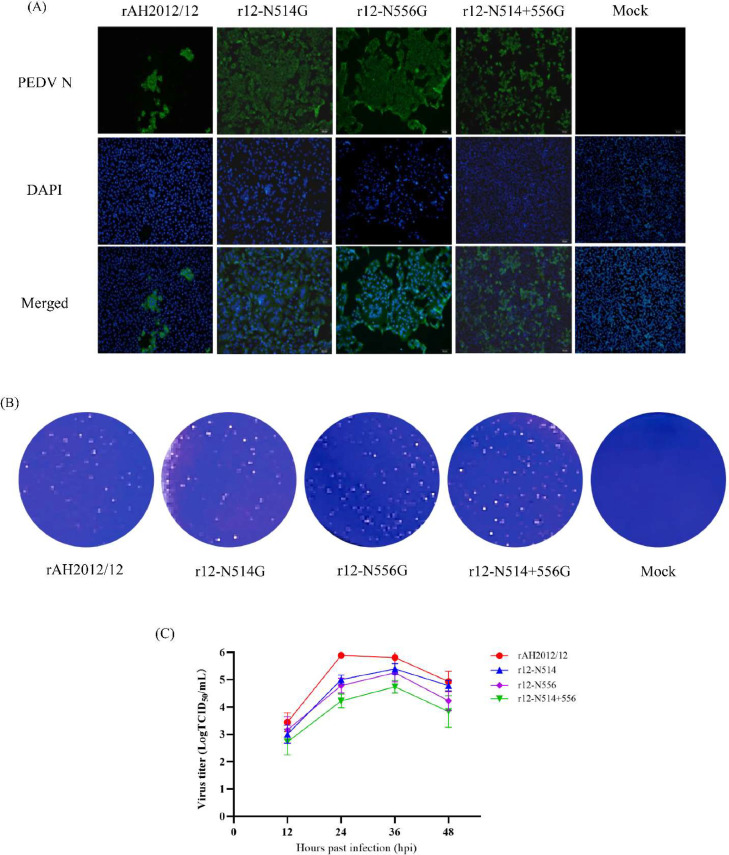
Characterization of the recombinant PEDVs. (A). Identification of the recombinant PEDVs by IFA. Scale bar, 200 μm. (B). Monolayers of Vero cells in 6-well plates were infected with PEDVs for 48 h, then the cells were fixed and stained with 0.1 % crystal violet. (C). Vero cells in 24-well plates were infected with 0.1 MOI of PEDVs, then the samples were collected at 12, 24, 36 and 48 hpi for titration with TCID_50_. Each time point was performed in triplicates.

Chimeric infectious cDNA clones (rPEDV-N514G, rPEDV-N556G, rPEDV-N514+556 G) were constructed through the reverse genetics system in our lab to remove the glycosylation sites in the RBD of spike protein (Fig. 1A). In the S protein RBD region, we substituted glutamine (G) with asparagine (N) at the 514th and 556th amino acid by homologous recombination to generate the recombinant BAC plasmids, pBAC-AH2012/12-N514G, pBAC-AH2012/12-N556G, and pBAC-AH2012/12-N514+556 G. Vero cells were transfected with BAC plasmids to rescue the recombinant PEDVs. As shown in Fig. 1, the recombinant PEDVs were successfully rescued by the evidence of sequencing of the mutation sites and IFA with mouse anti-PEDV N protein monoclonal antibody (Figs. 1B and 2A). While no IFA signal and CPE were observed in mock cells. These results indicated that we successfully constructed the recombinant PEDVs which depletion of glycosylation sites in the RBD.

### 3.2 Characterization of the recombinant PEDVs

After we obtained the recombinant PEDVs, the biological characteristics of these recombinant PEDVs were also determined in Vero cells. As shown in Fig. 2B, all the PEDVs can formed plaques in Vero cells, and the recombinant PEDVs shared similar plaque morphology with the parental PEDV rAH2012/12 (Fig. 2B). Based on growth kinetic results, all the PEDVs gradually increase its viral titer in Vero cells within 24 hpi, and the parental PEDV rAH2012/12 reached highest infectious titer (10^5.75^ TCID_50_/ml) at 24 hpi, but the recombinant PEDVs showed its highest viral titer at 36 hpi. The infectious titers for r12-N514G, r12-N556G, and r12-N514+556 G are 10^5.39^ TCID_50_/ml, 10^5.25^ TCID_50_/ml, and 10^4.74^ TCID_50_/ml, respectively (Fig. 2C). These results indicated that glycosylation in the RBD does not affect the plaque morphology but slightly decrease the virus titer in vitro.

### 3.3 Clinical sign and fecal virus shedding in piglets challenged with recombinant PEDVs

**Fig. 3:**
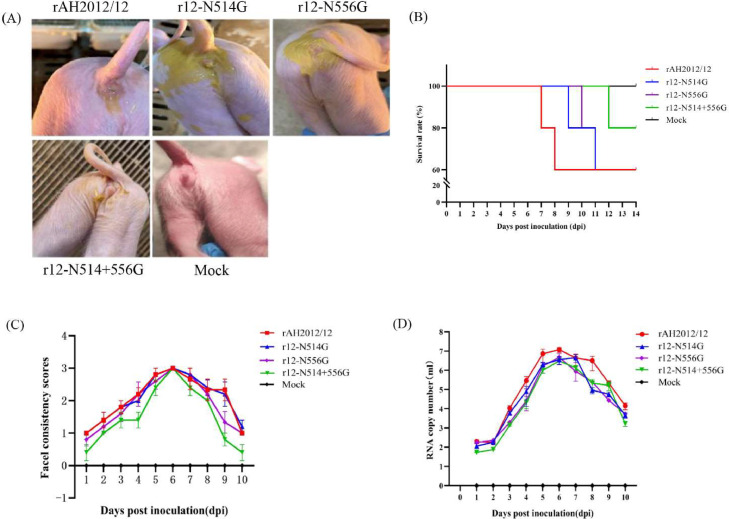
Clinical sign and fecal virus shedding in piglets challenged with the recombinant PEDVs. (A). Clinical evaluation of piglets following PEDV challenge. The photographs depicting symptoms of diarrhea in each experimental group were captured at 4 days post infection. (B). Survival rate of piglets challenged with different PEDV strains. (C). Fecal scores of piglets from different groups. fecal consistency scoring: 0 (solid), 1 (pasty), 2 (semiliquid), and 3 (liquid). (D). Fecal PEDV RNA shedding titers of each group within 10 dpi. Each symbol represents the titer of the PEDV N gene in 1 ml or 1 g of stool sample from an individual piglet collected daily. Each line indicates the mean values of a group.

To evaluate the pathogenicity of the recombinant PEDVs, rAH2012/12, r12-N514G, r12-N556G, r12-N514+556 G were introduced orally into five-day-old piglets with 1.0 × 10^4.5^ TCID_50_ of each indicated virus. Piglets from negative control showed no clinical signs during this study, and all were active and fleshy. In spite of the diarrhea and progressive dehydration, appetite was maintained. The rAH2012/12 group piglets began to show diarrhea until 3 dpi, whereas the other four groups developed diarrhea by 4 dpi. A 24 h delay in diarrhea development was seen between the piglets in the rAH2012/12 group and those in the other four groups (Fig. 3A). At 5–8 dpi, the challenged piglets developed lethargy, anorexia, diarrhea, and recovered gradually thereafter except for rAH2012/12 piglets which developed more severe symptoms. Notably, in the rAH2012/12-challenged group, one piglet died at 7 and 8 dpi, respectively. In the r12-N514G group, one piglet died at 9 and 11 dpi, respectively. In the r12-N556G group, one piglet was died at 10 and 11 dpi, respectively. one piglet died in r12-N514+556 G group at 12 dpi (Fig. 3B). Furthermore, it was observed that all pigs infected with the rAH2012/12, r12-N514G, and r12-N556G strains experienced moderate to severe diarrhea (diarrhea score ≥2) starting from 3 dpi. Conversely, the group infected with the r12-N514+556 G strain displayed diarrhea symptoms at 5 dpi. (Fig. 3C).

Virus shedding of the challenged piglets was also evaluated by RT-qPCR. The results demonstrated that the piglets in the challenged group exhibited significant viral shedding in their fecal samples, whereas the piglets in the normal control group tested negative for PEDV. The highest mean titers of PEDV RNA copy numbers in the rAH2012/12, r12-N556G, r12-N514+556 G groups were 10^7.07^ copies/ml, 10^6.66^ copies/ml, 10^6.70^ copies/ml at 6 dpi and r12-N514G group was 10^6.48^ copies/ml at 7 dpi (Fig. 3D).

### 3.4 An investigation into the histopathological lesions and distribution of the virus

**Fig. 4:**
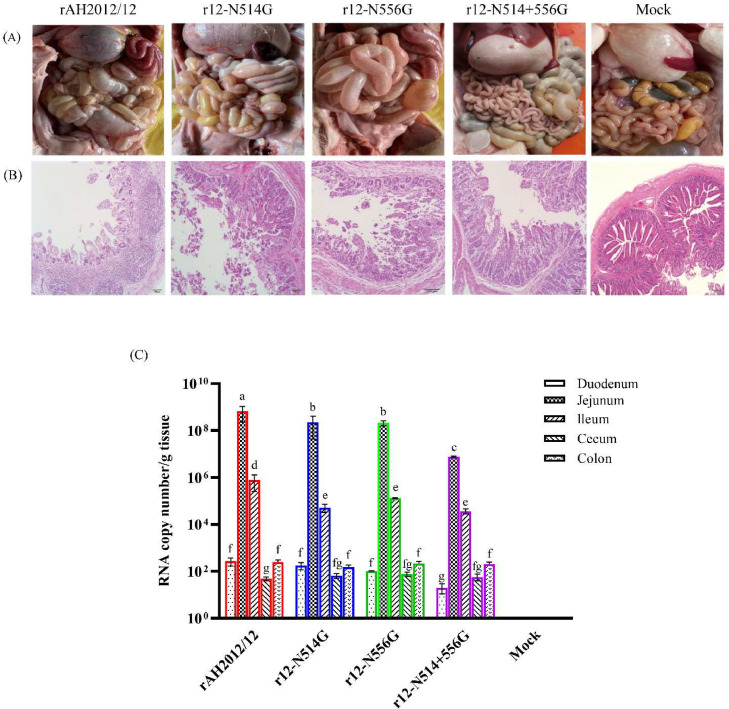
Histopathological lesions and virus distribution in piglets challenged with the recombinant PEDVs. (A). Macroscopic examinations of the intestine of piglets inoculated with rAH2012/12, r12-N514G, r12-N556G, r12-N514+556 G and DMEM medium, respectively. (B). HE-stained small intestines of piglets inoculated with different PEDV strains. (C). Quantification of viral loads in different intestine segments of all piglets per group. Different intestinal segments were sampled and tested for viral loads, and virus abundance was quantified by quantitative real-time RT-PCR targeting the PEDV N gene. The different letters indicate significant difference (*P* < 0.05), the same letters or no letter indicate no significant difference (*P* > 0.05).

A necropsy examination was performed after conducting the animal experiment. The piglets that were inoculated with the original and rescued viruses displayed characteristic symptoms resembling PED, which consisted of thin-walled small intestines containing soft to watery substances and distended stomachs filled with curdled and undigested milk (Fig. 4A). In contrast, there were no visible abnormalities in the other organs of the intestine. The histopathological analysis revealed that the jejunum of rAH2012/12-infected piglets displayed severe villous atrophy, while the jejunum of r12-N514G- and r12-N556G-infected piglets showed moderate villous atrophy. Interestingly, the piglets infected with r12-N514+556 G exhibited only mild pathological changes in jejunum, and no obvious pathological changes were detected in the controls (Fig. 4B). Following the animal experiment, the virus loads were determined in different intestinal segments of the inoculated piglets. PEDV was detected in all the intestinal segments of both the parent- and rescued-virus-infected piglets. Subsequent analysis revealed that the viral loads in the jejunum were significantly higher compared to the other intestinal segments in all the PEDV-infected groups (Fig. 4C).

### 3.5 Immunogenicity comparison between the recombinant PEDVs and the original PEDV

**Fig. 5:**
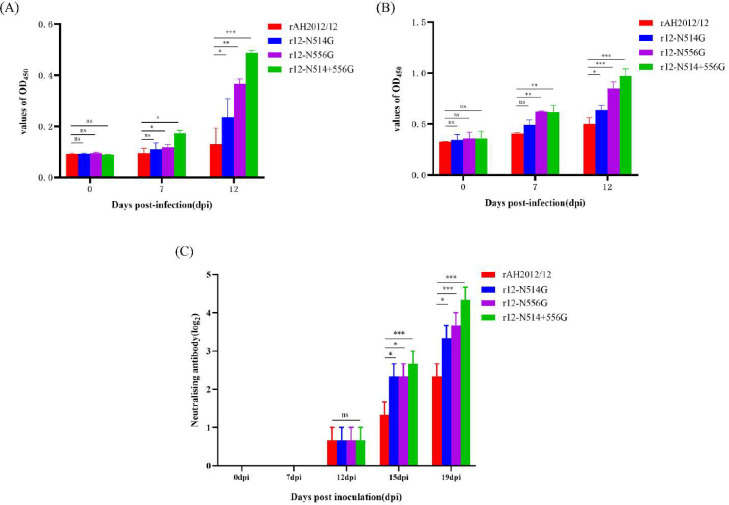
Immunogenicity comparison between the recombinant PEDVs and the original PEDV. (A). Serum IgA antibody levels in each challenged groups. (B). Serum IgG antibody levels in each challenged groups. (C). Neutralizing antibodies in serum from challenged piglets. Serum obtained at 0, 7, 12, 15, 19 dpi was measured by a serum neutralization test against PEDV AH2012/12. *** refers to extremely significant difference (*P* < 0.001). ** means this difference is statistically significantly different (*P* < 0.01). * denotes the presence of a statistically significant difference (*P* < 0.05). ns means the difference was not significant.

Serum specimens were collected at 0, 7, 12, 15, and 19 dpi, and the serum antibody responses induced by the parent and rescued PEDVs were assessed by ELISA and VN assays. The data presented in Fig. 5A and B demonstrate a consistent increase in levels of IgA and IgG antibodies among all experimental groups. The antibody titer reaching peak levels at 12 dpi. Notably, the r12-N514+556 G group exhibited the highest levels of IgA and IgG antibodies, while the antibody titers in the rAH2012/12 group were lower than those observed in the other challenged groups. Compared with the original PEDV, the recombinant PEDV-challenged groups showed higher antibody titers (*P* ≤ 0.05). Serum neutralization tests were conducted solely on ELISA-positive samples to assess neutralizing antibodies derived from the N-deglycosylated viruses. As depicted in Fig. 5C, no neutralizing antibodies were observed in any of the groups prior to inoculation, the neutralizing antibody induced by the parent- and rescued-virus were detected at 12 dpi. However, the antibody titers exhibited rapid increase thereafter. These reached the antibody titer of 1:10, 1:16, 1:25 at 19 dpi in piglets infected with r12-N514G, r12-N556G, r12-N514+556 G, which was higher than the titers in rAH2012/12-infected piglets (1:5).

## 4 Discussion

PEDV has been endemic since the 1970s in most countries. In 1980s, the pig industry has been perceived as being at risk from PEDV (Chen et al., 2008; Puranaveja et al., 2009). The emergence of highly virulent variant PEDV strains in China in 2010 resulted in the death of up to 100 % of pigs (Chen et al., 2011). Currently, PED is recognized as a pandemic disease, leading to significant economic losses for pork producers worldwide. Despite the extensive use of both inactivated and live-attenuated vaccines for controlling the predominance of PEDV in China and other countries, the ongoing PEDV outbreak persists, with the continual detection of novel PEDV strains in vaccinated pig herds (Carter et al., 2020; Wang et al., 2016). This was associated with inefficient protective immune induction against the currently prevalent PEDV strains. It is therefore urgent to develop candidate vaccine that can trigger high levels of neutralizing antibodies.

There is still no doubt that vaccinations can prevent outbreaks of PED. Various platforms have been devised for the development of vaccines against PED, including conventional strategies like inactivated and live-attenuated viruses, alongside contemporary techniques such as recombinant proteins and vectors. Of these, live attenuated vaccines exhibit high immunogenicity, typically requiring only a single immunization to induce protective immunity, thereby garnering significant attention. Up to date, some researchers has engineered PEDVs through reverse genetic systems, aiming to reduce viral virulence while maintaining high immunogenicity. These engineered strains, particularly those with modified RBD regions of the S protein, are considered potential candidates for the development of live attenuated vaccines against PEDV. This is due to the observed enhancement of immunogenicity through targeted mutations in this gene, as demonstrated in other coronaviruses like MHV and SARS-CoV (Du et al., 2009, 2017). The RBD of the S protein has the potential to elicit a robust neutralizing antibody response and provide cross-protection against other strains of coronaviruses. The utilization of RBD as an antigen has led to the isolation of numerous neutralizing antibodies that play a crucial role in the therapeutic treatment of PEDV. Furthermore, the RBD of coronaviruses also can bind to host receptor for entry. Currently, the functional receptor for PEDV is unknown. Although no experiment to prove RBD indeed plays a role in binding to the PEDV receptor, we can gain some implications from other swine coronaviruses, like porcine deltacoronavirus (PDCoV) and transmissible gastroenteritis virus (TGEV). The functional receptor for PDCoV and TGEV is aminopeptidase N (APN). A previous study by Ji et al. showed that the RBD of spike protein indeed binds to APN, and they also resolved the crystal structure of a deltacoronavirus RBD protein bound to porcine and human APN (Ji et al., 2022). In 2012, Reguera et al. described the crystal structures of RBD of TGEV in complex with their receptor, porcine APN (pAPN), and they found that conformation of the RBD determines cell entry receptor specificity (Reguera et al., 2012). Therefore, the RBD in the S protein is a an important target for vaccine development.

The change of N-glycosylation site of coronavirus S protein has been considered to be a key mutation affecting the infection, transmission, pathogenicity and immunogenicity of coronaviruses in mammals (Han et al., 2007). By introducing a mutation in the N-linked glycosylation site at position N44, a PRRSV isolate showed reduced production of neutralizing antibodies and resistance to antibody neutralization. (Faaberg et al., 2006). On the other hand, eliminating the N-linked glycan at N45 in LDV's GP5 greatly improved the immunogenicity of the neutralization epitope and increased susceptibility to antibody neutralization. (Chen et al., 2000). Mutations in seven N-glycosylation sites within the S protein of SARS-CoV-2 have important effects on the interaction with ACE2, which N227 and N699 enhance the transmission ability of the virus. Deletion of N331 and N343 glycosylation sites in the S protein of SARS-CoV-2 significantly reduced the infectivity of the virus (Bouwman et al., 2020; van Beurden et al., 2017). Deletion of N234 significantly increased virus antagonistic activity against neutralizing antibodies, while deletion of N165 enhanced the sensitivity of the virus to neutralizing antibodies (Li et al., 2020).

One previous study by Huang *et al*. quantitatively determined 18 of the 28 predicted N-glycosylation sites on PEDV PT52 spike protein through MS-based glycopeptide analysis (Huang et al., 2022). They identified these amino acid sites in the spike protein, including N62, N118, N216, N300, N344, N351, N556, and N667, contained terminal Hex-HexNAc units capped by an extra hex residue. Furthermore, cryo-EM maps showed clear protrusions at six sequons, Asn324, Asn514, Asn425, Asn726, Asn781, and Asn787, indicating these sites are also glycosylated (Huang et al., 2022). Based on above description, the sites, N514 and N556, are truly glycosylated in the RBD of spike protein of PEDV. So, this study chose the glycosylation sites, N514 and N556 in the RBD of spike protein, to evaluate the effects of glycosylation in RBD on the immunogenicity and pathogenicity of PEDV. The results showed that there was no significant difference between the parental and recombinant PEDVs in plaque morphology. From the growth curve of the virus in Vero, it can be seen that N514G, N556G, and N515+556 G slightly weakened the replication ability of AH2012/12 in Vero, reflecting that the mutant strains had a slight effect on the viral titer of AH2012/12. Furthermore, this may be due to recombination events occurring within and between different subgroups/species of CoV may be responsible for this phenomenon, as they are known to happen during genetic evolution (Li et al., 2020). Specifically, the genetic mutations N514G, N556G, and N515+556 G have been identified as causing genetic recombination, which in turn could result in a decrease in the replication capacity of the mutant. In animal experiments, both the parental strain and the mutant strain could cause tissue lesions, which detect the virus in the jejunum and feces. This may be due to slightly decreased replication of the virus *in vivo* after mutation, nevertheless, viral particles were not significantly reduced. However, compared with the parental virus, the replication efficiency of the recombinant virus could induce the production of highly effective IgG and neutralizing antibodies in piglets. We speculate that removal of glycosylation sites might expose an immunogenic epitope of the S protein to attenuate the virus capacity of evading the host immune response. Although these results suggests that the glycosylation sites in RBD do not affect the pathogenicity of PEDV, and the mutations in the N-glycosylation site in the RBD region of S protein enhance the immunogenicity of AH2012/12. Consequently, we suggest that altering the N-linked glycosylation site of the S protein could be a promising strategy for developing vaccine candidates that can induce high levels of neutralizing antibodies.

## 5 Conclusions

In this study, three recombinant PEDVs were engineered to evaluate the effects of glycosylation in RBD on the pathogenicity and immunogenicity of PEDV. The glycosylation sites in RBD do not affect the plaque morphology, but slightly reduce the infectious titers of PEDV AH2012/12. The RBD glycosylation has no influence on the pathogenicity but can attenuate the immunogenicity of PEDV AH2012/12. These data provide basis for design and production of vaccines against PED.

## CRediT authorship contribution statement

**Gege Zhang:** Methodology, Validation, Data curation, Investigation, Writing – original draft. **Qi Peng:** Validation, Data curation, Writing – original draft, Writing – review & editing. **Shiyu Liu:** Investigation. **Baochao Fan:** Formal analysis, Investigation, Supervision. **Chuanhong Wang:** Investigation. **Xu Song:** Investigation. **Qiuxia Cao:** Investigation. **Chengcheng Li:** Investigation. **Hong Xu:** Investigation. **Hongting Lu:** Investigation. **Meiying Bao:** Investigation. **Shanshan Yang:** Investigation. **Yunchuan Li:** Investigation. **Jiaxiang Wang:** Conceptualization, Writing – review & editing. **Bin Li:** Conceptualization, Methodology, Validation, Resources, Data curation, Formal analysis, Writing – review & editing, Visualization, Supervision, Project administration, Funding acquisition.

## Declaration of competing interest

The authors have no conflicting interests.

## Data Availability

Data will be made available on request.
